# TEDDI: radiotherapy delivery in deep inspiration for pediatric patients − a NOPHO feasibility study

**DOI:** 10.1186/s13014-018-1003-4

**Published:** 2018-03-27

**Authors:** Anni Young Lundgaard, Lisa Lyngsie Hjalgrim, Laura Ann Rechner, Mirjana Josipovic, Morten Joergensen, Marianne Camille Aznar, Anne Kill Berthelsen, Lise Borgwardt, Christoffer Johansen, Annika Loft, Akmal Safwat, Leila Vaalavirta, Lena Specht, Maja Vestmoe Maraldo

**Affiliations:** 1 Department of Clinical Oncology, Rigshospitalet, University of Copenhagen, Blegdamsvej 9, 2100 Copenhagen, Denmark; 2 Department of Pediatric Haematology and Oncology, Rigshospitalet, University of Copenhagen, Blegdamsvej 9, 2100 Copenhagen, Denmark; 3 Niels Bohr Institute, University of Copenhagen, Blegdamsvej 17, 2100 Copenhagen, Denmark; 4 Manchester Cancer Research Centre, Division of Cancer Sciences, School of Medical Sciences, Faculty of Biology, Medicine and Health, University of Manchester c/o Christie Hospital, Department 58, Floor 2A, Wilmslow Road, Manchester, M20 4BX UK; 5 Department of Clinical Physiology, Nuclear Medicine and PET, Rigshospitalet, University of Copenhagen, Blegdamsvej 9, 2100 Copenhagen, Denmark; 6 Danish Cancer Society Research Center, Strandboulevarden 49, 2100 Copenhagen, Denmark; 7 Department of Clinical Oncology, Aarhus University Hospital, Palle Juul-Jensens Boulevard 99, 8200 Aarhus, Denmark; 8 Department of Radiation Oncology, Comprehensive Cancer Center, Helsinki University Hospital, Haartmaninkatu 4, 00290 Helsinki, Finland

**Keywords:** Deep inspiration breath-hold, Pediatric patients, Radiotherapy

## Abstract

**Background:**

Radiotherapy (RT) delivered in deep inspiration breath-hold (DIBH) is a simple technique, in which changes in patient anatomy can significantly reduce the irradiation of the organs at risk (OARs) surrounding the treatment target. DIBH is routinely used in the treatment of some adult patients to diminish the risk of late effects; however, no formalized studies have addressed the potential benefit of DIBH in children.

**Methods/Design:**

TEDDI is a multicenter, non-randomized, feasibility study. The study investigates the dosimetric benefit of RT delivered in DIBH compared to free breathing (FB) in pediatric patients. Also, the study aims to establish the compliance to DIBH and to determine the accuracy and reproducibility in a pediatric setting. Pediatric patients (aged 5–17 years) with a tumor in the mediastinum or upper abdomen with the possible need of RT will be included in the study. Written informed consent is obligatory. Prior to any treatment, patients will undergo a DIBH training session followed by a diagnostic PET/CT- or CT-staging scan in both DIBH and FB. If the patient proceeds to RT, a RT planning CT scan will be performed in both DIBH and FB and two separate treatment plans will be calculated. The superior treatment plan, i.e. equal target coverage and lowest overall dose to the OARs, will be chosen for treatment. Patient comfort will be assessed daily by questionnaires and by adherence to the respiratory management procedure.

**Discussion:**

RT in DIBH is expected to diminish irradiation of the OARs surrounding the treatment target and thereby reduce the risk of late effects in childhood cancer survivors.

**Trial registration:**

The Danish Ethical Committee (H-16035870, approved November 24th 2016, prospectively registered). The Danish Data Protection Agency (2012–58-0004, approved January 1st 2017, prospectively registered). Registered at clinicaltrials.gov (NCT03315546, October 20th  2017, retrospectively registered).

## Background

Long-term survival following childhood cancer is excellent, with 5-year overall survival rates exceeding 80% [1, 2]. It is, however, thoroughly documented that childhood cancer survivors suffer from a treatment-induced excess mortality and morbidity when compared to the general population or siblings [3–5].

Radiotherapy (RT) is known to induce late effects in childhood cancer survivors; primarily cardiovascular disease and second cancers [5–10]. Due to the long latency period, radiation-induced late effects are difficult to assess and quantify as they are often the consequence of treatment regimens now considered outdated. Nonetheless, the risk of radiation-induced late effects is known to be influenced by both the radiation dose and the volume of irradiated tissue [7, 11–13].

In modern RT, advanced imaging, highly conformal treatment planning and delivery techniques, as well as respiratory motion management systems have been introduced in the adult setting in order to reduce the radiation to healthy organs at risk (OARs) surrounding the treatment target. Deep inspiration breath-hold (DIBH) is a simple technique where the irradiation is delivered only while the patient holds his/her breath. A breath-hold in deep inspiration causes a change in patient anatomy (i.e. inflation of the lungs, rotation and elongation of the heart) and imaging artifacts from respiratory movement are diminished. The technique significantly reduces the radiation dose to the surrounding OARs, without compromising the delivered radiation dose to the treatment target, and with no detriment to other healthy organs [14–17]. The OAR dose reduction is estimated to reduce the risk of late effects [18]. Consequently, DIBH is widely used across the world for adult patients with left-sided breast cancer and mediastinal lymphoma.

However, the experience with DIBH in the pediatric setting is sparse [19, 20]. In general there has been a reluctance to implement new RT techniques in the pediatric setting as late effects data are not available with these new techniques. Regarding motion management systems an additional concern has been the skepticism about children’s compliance with breathing instructions.

With TEDDI we will introduce radio**t**h**e**rapy **d**elivery in **d**eep-**i**nspiration for pediatric patients within a multicenter setting through the NOPHO (Nordic Society of Pediatric Haematology and Oncology) network; accommodating the need for systematic research of RT delivery techniques in pediatric patients.

## Methods/Design

### Aims and hypothesis

TEDDI aims to

1. Estimate the dosimetric benefit of RT using DIBH compared to free breathing (FB) in pediatric patients.

*Hypothesis 1: For more than 75% of patients, treatment in DIBH will be dosimetrically superior to treatment in FB.*

2) Establish the compliance of DIBH in pediatric patients.

*Hypothesis 2: Over 90% of pediatric patients from the age of five years will be able to perform stable, reproducible, and comfortable DIBHs (of 20 seconds) through their course of RT.*

3) Determine if DIBH is an accurate and reproducible strategy for pediatric patients.

*Hypothesis 3: The tumor position will be reproducible from day to day, as well as from DIBH to DIBH. Variations in tumor position will be less than 5 mm over the whole treatment course.*

### Patients

25 consecutive, pediatric cancer patients will be included.

Inclusion criteria:

- Age 5–17 years.
- Patients with a tumor in the mediastinum or upper abdomen with the possible need for RT according to current treatment guidelines, irrespective of cancer diagnosis.
- The ability to perform three sequential DIBHs of 20 s each during a training session.
- Written informed consent from parent(s) or legal guardian(s).

Exclusion criteria

- Age < 5 years or > 17 years at time of diagnosis.
- The need for sedation during RT.
- CNS tumor or pelvic localization.
- Unable to understand DIBH coaching information directly or through interpretation.

### Patient information

Patient and parent(s) will receive written and oral information about the protocol and be accrued prior to start of any treatment (chemotherapy or surgery) at the Pediatric Department.

### Training session

**Fig. 1:**
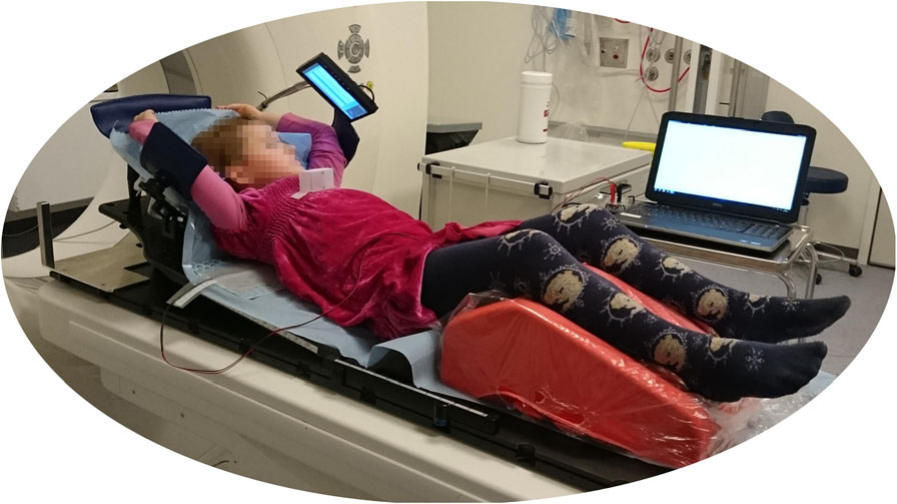
A six year old healthy volunteer perform deep inspiration breath-hold using the Real-time Position Management system from Varian Medical Systems (Palo Alto, USA). The screen provides a visual feed-back and helps guide the volunteer’s  respiration. Notice how the immobilization device does not support the volunteer sufficiently; if the volunteer was to receive radiotherapy a custom made fixation device should be constructed

The patient and parent(s) receive oral and written instruction on how to perform DIBHs. The information is adjusted to the individual patient’s age. At Rigshospitalet the Real-time Position Management (RPM) system from Varian Medical Systems (Palo Alto, USA) is used to perform and monitor DIBHs (during training, scanning, and treatment) [21]. The system consists of a small plastic marker box which is placed on the patient’s thoracic wall. The depth of inspiration is followed by an infra-red camera and expressed by the anterior-posterior displacement of the marker box. The patient is given visual feedback from a video screen which illustrates the level of inspiration; cf. Fig 1. Non-respiratory movement is detected through careful visual observation of the patient.

The patient is deemed compliant if he/she successfully completes three sequential DIBHs of 20 s each during the 20 min training session.

### Treatment planning

All diagnostic imaging during treatment and treatment planning will be performed according to national guidelines (as per cancer diagnosis). For optimal RT planning, the patient must be scanned upfront (pre-chemotherapy and/or pre-surgery) in the treatment position (i.e. in the supine position with arms raised over the head) in both DIBH and FB. This way the uncertainties in the image registration and fusion between the upfront imaging and imaging for treatment planning are minimized. Whenever a positron emission tomography (PET) scan is considered an integral part of the RT planning, a PET scan should be performed upfront as well [22]. The additional PET/computed tomography (CT) scan in DIBH can be conducted as a limited one-bed PET/CT scan over the mediastinal region, preferably in a joint session with the staging PET/CT scan. However, for institutions where this is not feasible, participation in TEDDI is still possible, and an upfront CT scan in both DIBH and FB will be sufficient.

For RT planning, a planning CT scan will be performed in DIBH as well as in FB for each patient.

Two RT plans will be made based on information from both pre- and post-chemotherapy DIBH and FB CT scans following international guidelines [23, 24]. On both scans the gross tumor volume (GTV), the clinical target volume (CTV), and the planning target volume (PTV) will be delineated. The PTV margin is left at the discretion of the treating institution and will be related to the specific positioning and image guidance strategies for each treatment site. All relevant OARs within the irradiated volume will be contoured (e.g. heart, female breasts, lungs, esophagus, thyroid, salivary glands, spinal cord, bone marrow, stomach, spleen, kidneys, and liver). The RT plan in DIBH and in FB will be calculated, both with similar planning objectives for the treatment target and OARs. All DIBH treatment plans will be designed to keep the number of breath-holds per fraction as low as reasonably achievable (including image guidance) for patient comfort.

### Treatment delivery

Patients will be treated in DIBH if the treatment plan in DIBH is superior to the treatment plan in FB with respect to the lowest overall dose to the OARs while maintaining acceptable target coverage. Coverage of the CTV and PTV will have the highest priority, as per ICRU83 guidelines [25]. The prioritizing of different OARs as well as the final choice of treatment plan will be at the discretion of the treating radiation oncologist.

To ensure the accuracy of the treatment delivery, image guided RT will be employed using daily volumetric imaging, planar kV imaging or surface imaging, depending on target location and institution.

**Fig. 2:**
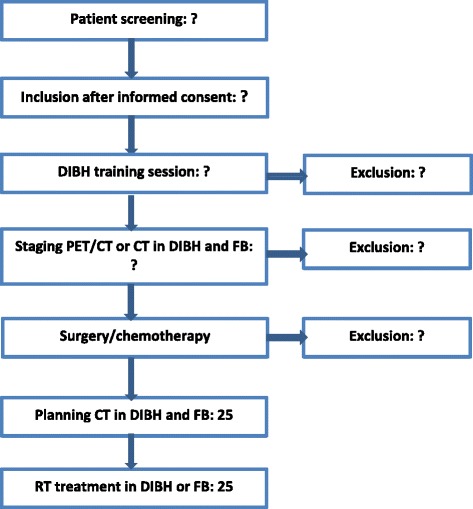
Chart of estimated workflow during accrual and treatment planning. *Abbreviations:* CT = computed tomography; DIBH = deep inspiration breath-hold; FB = free breathing; PET = positron emission tomography; RT = radiotherapy

Cf. Fig 2 for a display of estimated workflow during accrual and treatment planning.

### Follow-up

Enrollment in TEDDI will not affect the standard follow-up program of pediatric patients, which is diagnosis specific.

### Safety

Procedures for patient safety and quality assurance during RT will be adhered to, irrespective of treatment in DIBH or FB. Patient compliance will be assessed through a questionnaire after each delivered RT fraction. Patients that initially or at any point during treatment cannot comply with the DIBH procedure will be treated in FB. A change from DIBH to FB must not cause a delay in the patient’s treatment course.

### Ethical considerations

Patient consent files will be kept at the local institutions. All data (e.g. clinical report forms, imaging, and radiation treatment information) will be anonymized, and saved for a period of ten years. All data analysis will be performed in Copenhagen.

Patients participating in TEDDI will be exposed to an additional radiation of approximately 5 mSv originating from the additional staging PET/CT scan (or CT scan) in DIBH. This corresponds to approximately 1.5 years of exposure to the natural background irradiation. Patients who are referred to RT will receive additional approximately 10 mSv from the additional planning CT scan in DIBH. We expect the potential benefits of DIBH in terms of a reduced dose to the heart and lungs and possibly also other organs will markedly outweigh this additional risk.

### Data analysis

TEDDI is a non-randomized feasibility study and for this mainly descriptive statistics will be applied. The patient cohort will be described by demographic data, histological diagnosis, stage, age, and gender. For each patient, target volumes (GTV, CTV, and PTV) and OARs volumes of the two planning CT scans will be compared. Maximum and mean doses as well as the volumes exposed to 5/10/20/30 Gy will be compared. The Wilcoxon signed rank test for paired data will be used for statistical testing.

The dosimetric parameters are surrogate markers for the potential clinical benefit with DIBH compared to FB RT plans as an observed, significant reduction in treatment-induced late effects would require several decades of follow-up in a patient cohort consisting of several hundreds of patients. The expected clinical implications will, however, be estimated by applying normal tissue complication probability-models derived from clinical case-control and cohort studies.

Patient compliance is defined as patient comfort and DIBH reproducibility. Hence compliance will be assessed using both qualitative and quantitative measures. Patient comfort will be assessed through daily questionnaires based on a 5-point Likert scale. Tumor position will be used to evaluate the ability of patients to maintain the breath-hold during the whole course of treatment and to investigate the possibility that patients might get weaker and/or more tired and thus unable to perform a stable DIBH as the treatment course progresses. This will be done using the respiratory motion management system.

The accuracy of the treatment delivery will be evaluated based on daily imaging. If the target is not visible, the sternum (mediastinal tumors) or the diaphragm (abdominal tumors) will be used as a surrogate structure. If the target position appears reproducible within 5 mm, the patient will be deemed compliant. In addition to the daily online positioning at the treatment machine before each fraction, the reproducibility of the target position will be assessed retrospectively by a medical physicist on a weekly basis.

### Sample size

Sample size is calculated based on a presumed mean heart radiation dose of 4 Gy in FB and a 25% reduction in mean heart dose with treatment in DIBH, with a standard deviation of 1.5 Gy across the patient group [16]. Recruiting 22 patients would lead to a power of 0.85 to detect this dosimetric difference. The Type 1 error probability is set at 0.05.

### Collaborators

TEDDI is open for accrual in Denmark. However, in order to ensure a faster accrual as well as a higher patient number to demonstrate uniformity across cancer diagnoses participation in TEDDI is planned within the NOPHO network in Sweden and Finland (Helsinki, Kuopio, Oulu, Turku, and Tampere).

## Discussion

The dosimetric benefit from RT in DIBH has been well documented in the adult setting [14–17], however respiratory motion management is not routinely used in the pediatric setting.

TEDDI is the first formalized study to introduce RT delivery in DIBH for pediatric patients.

Prior to clinical implementation of TEDDI, a pilot study was conducted at Rigshospitalet in order to investigate whether children from the age of five are able to perform stable and reproducible DIBHs using the RPM system. To assess the patient compliance qualitatively, the participating children and families completed a questionnaire after the DIBH training session. The questionnaire evaluated the child’s comfort with the pre-training information and the DIBH coaching. Based on experiences gained from this pilot study a standard operating procedure for RT delivery in DIBH for pediatric patients has been developed.

Two prior studies have described the experience with DIBH in pediatric patients [19, 20]. Both studies used a spirometry-assisted breath-hold technique. Claude et al. [19] concluded that only older children are able to comply and understand the technique, and it increases the estimated daily treatment time at the linac. Spirometry-assisted DIBH has in adults been reported less comfortable than the optical surface tracking DIBH approach [26], employed in TEDDI. Our DIBH-method is performed as a non-invasive procedure and constitutes a very gentle treatment expected to cause a minimal inconvenience to this fragile patient population. When treating very young children, however, prolonged treatment time at the linac might be unavoidable.

Recently Huijskens et al. investigated the respiratory-induced motion of the diaphragm and the intra- and interfractional variability in children during image-guided RT [27]. They found a large range of the diaphragm amplitude motion, mean 10.7 mm (range 4.1–17.4 mm), with the intrafractional variability being the largest. Because of the substantial patient variability they concluded that a 4DCT in children should be performed to quantify the individual respiratory motion in order to define individual safety margins. TEDDI will clarify whether RT in DIBH can reduce this variability, potentially allowing for smaller margins and, thus, a reduced dose to the OARs.

DIBH seems to be compatible with proton therapy, however, data and availability is still limited. The experience gained from TEDDI will be directly transferable to proton treatment facilities. Also, DIBH holds the potential for synergistic combinations with other new RT principles, e.g. dose painting with a boost to radio-resistant areas of the tumor, as this technique requires that tumor motion is minimal in order to deliver the radiation dose correctly.

In conclusion, RT in DIBH is a promising technique in a pediatric setting, which could have a dramatic impact on the risk of late effects following RT for childhood cancer, improving subsequent quality of life, morbidity, and - ultimately - mortality.

## Abbreviations

CT: Computed tomography
CTV: Clinical target volume
DIBH: Deep inspiration breath-hold
FB: Free breathing
GTV: Gross tumor volume
NOPHO: Nordic Society of Pediatric Haematology and Oncology
OARs: Organs at risk
PET: Positron emission tomography
PTV: Planning target volume
RPM: Real-time Position Management system
RT: Radiotherapy
TEDDI: Radiotherapy delivery in deep inspiration for pediatric patients
